# Genome sequence of *Sulfurimonas* sp. C5, a potential chemolithoautotrophic, sulfur-oxidizing bacterium isolated from a mangrove sediment

**DOI:** 10.1128/mra.00474-24

**Published:** 2024-09-24

**Authors:** Qing Yuan, Shufang Wang, ShaSha Wang, Yangsheng Zhong, Lijing Jiang

**Affiliations:** 1 Key Laboratory of Marine Genetic Resources, Third Institute of Oceanography, Ministry of Natural Resources of PR China; Fujian Key Laboratory of Marine Genetic Resources, Xiamen, China; 2 School of Marine Science and Fisheries, Jiangsu Ocean University, Lianyungang, China; University of Southern California, Los Angeles, California, USA

**Keywords:** sulfur-oxidizing, chemolithoautotroph, mangrove sediment

## Abstract

*Sulfurimonas* sp. strain C5 (MCCC 1A19556) is a strain with potential chemolithoautotrophic sulfur-oxidizing function, which is isolated from a mangrove sediment sample collected from Quanzhou Bay, Fujian Province, China. This report describes the genome sequence of strain C5, which possesses the gene sets for the sulfur-oxidizing pathway.

## ANNOUNCEMENT

Members of genus *Sulfurimonas* belonging to phylum *Campylobacterota* are ubiquitous in diverse environments and especially dominant in deep-sea and shallow hydrothermal vents (1–4), cold seeps (5), and marine sediments (6–8). *Sulfurimonas* sp. strain C5 was isolated from the mangrove sediment (a depth of 20 cm) collected at Quanzhou Bay, Fujian Province (24°93′N, 118°40′E). The 2-g sediment samples were pre-mixed in sterilized natural seawater, and then 1 mL was transferred to 50-mL serum bottles with 10 mL of MMJHS liquid medium according to a previous protocol (9). For the growth of strain C5, both the reduced sulfur compounds (S_2_O_3_^2-^) and H_2_ were used as electron donors, and O_2_ served as the electron acceptor. After 2 days of incubation, significant cell growth was observed in the MMJHS medium. Subsequently, cells were purified with the dilution-to-extinction technique (10). After repeating the dilution-to-extinction three times from the well-grown culture at the maximum dilution series (10^–7^), a pure culture was finally obtained and confirmed by the microscopic observation and 16S rRNA gene sequencing. Pure cultures after dilution-to-extinction were inoculated 1 mL into 10 mL of the MMJHS medium. After 24 hours of incubation , the total DNA of strain C5 was extracted, and the 16S rRNA gene was amplified. Primers used for PCR were 27F(5′-AGAGTTTGATCCTGGCTCAG-3′) and 1492R(5′-TACGGTTACCTTGTTACGACTT-3′). Similarity of 16S rRNA gene sequences was determined using sequence comparison in the EzBioCloaud server (http://www.ezbiocloud.net/) and BLASTN in the NCBI database.

The genome sequence of strain C5 was determined by Shanghai Majorbio Bio-pharm Technology Co., Ltd. (Shanghai, China). Paired-end libraries (insert size, 712 bp) were prepared using a VAHTS universal DNA library prep kit for Illumina. Sequencing was performed on the Illumina NovaSeq 6000 platform (2 × 150 bp), and a paired-end library with a total of 4,457,883*two read pairs was obtained. Raw reads obtained after sequencing were filtered using a fastp software (version 0.19.6) (11), followed by assembly with SOAPdenovo2.04 (12), and these procedures ultimately yielded genome sequences for the strain C5. The prediction of coding sequences (CDS) in the genome was carried out using NCBI Prokaryotic Genome Annotation Pipeline (PGAP) (version 6.5) (13). In addition, the identification of rRNA and tRNA genes was conducted using the Barrnap GPLv3 (https://github.com/tseemann/barrnap) and tRNAscan-SE v2.0 (14), respectively. The average nucleotide identity (ANI) values between C5 and its relatives were estimated using OrthoANI computation (http://www.ezbiocloud.net/tools/ani) (15). Digital DNA–DNA hybridization (dDDH) were performed on the GGDC website (https://ggdc.dsmz.de/). Default parameters were used for all software, unless otherwise specified.

C5 had a genome size of 2,064,714 bp with 27 contigs (N_50_ value, 343,974 bp; G + C content, 34.86 mol%; coverage, 100×). A total of 2,132 genes were predicted as six rRNAs, 43 tRNAs, and 2,074 protein-coding sequences in the C5 genome (Table 1). The ANI and dDDH values between the strain C5 and its closest relative *Sulfurimonas marina* B2 (CP041165) were 80.31% and 22.7%, respectively. BLAST analysis of the 16S rRNA gene sequences indicated that strain C5 shared the highest sequence similarity (97.96%) with *Sulfurimonas marina* B2 (MN069316). Taken together with above data, strain C5 could be a representative of a new species of the genus *Sulfurimonas*.

**TABLE 1:** Statistics for genome assemblies of *Sulfurimonas* sp. C5

Characteristic	Data
Size (bp)	2,064,714 bp
Contig	27
Coverage(×)	100
G + C content (%)	34.86%
N_50_ value (bp)	343,974 bp
Number of predicted CDSs	2,074
Genes assigned to COG	1,693 (81.1%)
Genes assigned to KEGG	1,278 (61.2%)
Number of rRNA genes	2, 2, 2 (5S, 16S, 23S)
Number of tRNAs	43
Genomic islands	3
GenBank accession no.	JARXNQ000000000

## Data Availability

The GenBank accession numbers for the 16S rRNA gene sequence, genome sequence, and sequence read archive of *Sulfurimonas* sp. C5 are OQ692571, JARXNQ000000000, and SRR24075631, respectively. The strain has been submitted to Marine Culture Collection of China, MCCC (http://www.mccc.org.cn/) with the accession number MCCC 1A19556.
